# Aqueous extract of *Salvia miltiorrhiza Bunge* reduces blood pressure through inhibiting oxidative stress, inflammation and fibrosis of adventitia in primary hypertension

**DOI:** 10.3389/fphar.2023.1093669

**Published:** 2023-02-28

**Authors:** Ruoyu Wu, Yongjie Zhou, Hongjun Xu, Wei Zhao, Luyang Zhou, Yilin Zhao, Qingzhuo Cui, Junda Ning, Hongxu Chen, Shengjun An

**Affiliations:** ^1^ Hebei Provincial Engineering Laboratory of Plant Bioreactor Preparation Technology, Hebei University of Chinese Medicine, Shijiazhuang, Hebei, China; ^2^ College of Integrated Chinese and western medicine, Hebei University of Chinese Medicine, Shijiazhuang, Hebei, China; ^3^ Medical College, Hebei University of Engineering, Handan Economoc and Technological Development Zone, Handan, Hebei, China

**Keywords:** spontaneously hypertensive rats, vascular adventitia, inflammation, oxidative stress, fibrosis, aqueous extract of *Salvia miltiorrhiza*

## Abstract

**Background:** Hypertension is a major risk factor for cardiovascular diseases and the leading cause of mortality worldwide. Despite the availability of antihypertensive drugs, alternative treatments are needed due to the adverse events associated with their use. Previous studies have shown that SABP, a combination of aqueous active metabolites of *Salvia Miltiorrhiza Bunge* DSS, Sal-A, Sal-B and PAL, has a significant antihypertensive effect. However, the underlying mechanisms remain unknown.

**Objective:** This study aimed to determine the effects of SABP on vascular inflammation, oxidative stress, and vascular remodeling in spontaneously hypertensive rats (SHRs). Additionally, the response of adventitial fibroblasts in SHRs to SABP treatment was also studied, including their proliferation, differentiation, and migration.

**Methods:** SABP or perindopril (positive control) were administered intraperitoneally to SHRs, and systolic blood pressure was measured using a tail-cuff approach. The effects of SABP on oxidative stress, inflammation, and vascular remodeling were investigated by transmission electron microscopy, histochemical staining, and Western blot. Adventitial fibroblasts were isolated and cultured from the adventitia of thoracic aorta in SHR and WKY rats. CCK8 assay, wound healing method and immunostaining were used to observe the effect of SABP on fibroblasts proliferation, migration and transformation into myofibroblasts. Moreover, Western blot analysis was also performed to detect the proteins related to oxidative stress, inflammation and fibrosis in adventitial fibroblasts.

**Results:** SHRs displayed higher blood pressure with significant vascular remodeling compared to WKY rats. The thoracic aorta and adventitial fibroblasts of SHRs exhibited significant oxidative stress, inflammation and fibrosis. SABP treatment repressed oxidative stress, inflammatory reaction and vascular remodeling of thoracic aorta in SHR through the ROS/TLR4/NF-κB signaling pathway, and inhibited fibrosis of thoracic aorta. Additionally, SABP inhibited the proliferation and migration of adventitial fibroblasts and their transformation to myofibroblasts *in vitro* through the TGFβ/Smad3 signaling pathway.

**Conclusion:** These findings suggest that SABP have potential as an alternative treatment for hypertension by ameliorating oxidative stress, inflammation and fibrosis. Further research is needed to fully understand the mechanisms underlying the effects of SABP.

## Graphical Abstract

**Figure:**
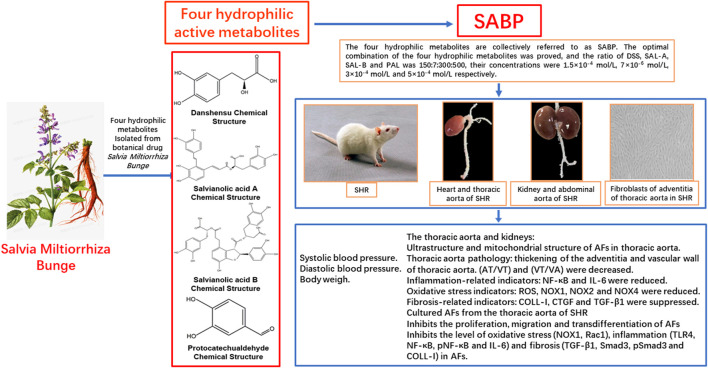
SABP, an optimized compatibility of four hydrophilic metabolites (DSS, Sal-A, Sal-B and PAL) from *Salvia miltiorrhiza Bunge*, can reduce the systolic blood pressure in SHR through its antioxidant, anti-inflammatory, anti-vascular remodeling and anti-fibrosis effects.

## Introduction

Hypertension is often considered an easily treated chronic non-communicable disease because of its simple blood pressure measurement and low-cost drug regimen (Angell et al., 2015). However, the most common adverse events associated with antihypertensive drugs, such as perindopril, an ACE inhibitor, are peripheral edema, cough, headache and dizziness (Shirley and McCormack, 2015), which highlights the need for alternative treatment. A recent study has found that hypertension is actually a secondary atherosclerotic complication resulting from increased serum cholesterol, rather than a primary disease. Additionally, hypertension exacerbates the severity of atherosclerosis (Hurtubise et al., 2016). No hypertension treatment trial has provided evidence that lowering blood pressure can reduce the risk of hypertensive atherosclerosis (Doyle, 1991). In the pathogenesis of atherosclerosis at the cellular and molecular levels is characterized by monocyte macrophages, oxidative stress, low-density lipoprotein modification, inflammatory mediators, and the influence of local hemodynamic environment (Kattoor et al., 2019), which results in abnormal changes in the structure and function of vascular walls. This is the main pathological basis of cardiovascular diseases such as atherosclerosis and hypertension (Zhang et al., 2021; Majesky and Weiser-Evans, 2022). The adventitia is the most complex section of the vessel wall and is comprised of various cells, including fibroblasts. These adventitial fibroblasts are often the first to be activated in response to vascular stress or injury (Stenmark et al., 2013). And play an important role in remodeling through proliferation, migration and production of ROS, cytokines and growth factors (Touyz and Schiffrin, 2004; Haurani and Pagano, 2007; Li et al., 2021). Inflammatory cells also contribute to oxidative stress by producing ROS through the phagocytic NADPH oxidase. Oxidative stress and fibrosis are two common molecular mechanisms that coordinate cellular events and organ systems in hypertension (Harvey et al., 2016; Touyz et al., 2020). Traditional Chinese medicine may serve as a complementary and alternative approach to the prevention and treatment of cardiovascular disease (Hao et al., 2017), but the underlying mechanisms are not well understood.

In this study, the effects of the active metabolites of *Salvia miltiorrhiza Bunge*, traditional Chinese medicine, on oxidative stress, inflammation and fibrosis were investigated using spontaneously hypertensive rats (SHR) and primary cultured adventitial fibroblasts (AFs) isolated from adventitia of aorta in SHR. *Salvia miltiorrhiza Bunge*, produced in Tangshan, China, is a commonly used herb in traditional Chinese medicine for decades and has been used to treat numerous disorders, particularly coronary heart diseases and cerebrovascular diseases (Su et al., 2015). The active metabolites of *S. miltiorrhiza Bunge* are hydrophilic include danshensu (DSS), salvianolic acid A (Sal-A), salvianolic acid B (Sal-B) and protocatechuic aldehyde (PAL), which combination is referred to as SABP. DSS [3-(3, 4-dihydroxy-phenyl) lactic acid] is structurally composed of a catechol and a lactic acid, and the catechol is the main active group that exerts an antioxidation effect against cardiac ischemia/reperfusion injury (Zhang et al., 2019). Similarly, Salvianolic acids have been reported to exerts cardio-protection in animal models of acute myocardial infarction (Yu et al., 2017). In addition, PAL potently protects cardiomyocytes through antioxidant activity (Jiang et al., 2019). However, the mechanisms of SABP, the optimal combination of hydrophilic active metabolites of *S. miltiorrhiza Bunge*, in antioxidation, anti-inflammation and anti-fibrosis *in vivo* and *in vitro* are not fully known. This study aimed to demonstrate the underlying mechanisms the antioxidant, anti-inflammatory and anti-fibrotic effect of SABP in SHRs and primary cultured Afs.

## Material and methods

### Chemicals and reagents

**FIGURE 1:**
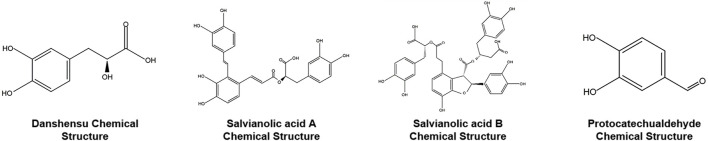
The chemical structures of the main metabolites identified in the aqueous extract of *Salvia miltiorrhiza Bunge*.

The four main hydrophilic metabolites of *S. miltiorrhiza Bunge* are DSS (Danshensu), Sal-A (Salvianolic Acid A), Sal-B (Salvianolic Acid B) and PAL (Protocatechuic aldehyde), collectively referred to as SABP. DSS (Cat. No. HY-N1913, Purity (NMR)≥98.0%), Sal-A (Cat. No. HY-N0318, Purity (HPLC): 99.75%) and Sal-B (Cat. No. HY-N1362, Purity (LCMS): 99.92%) were purchased from MedChemExpress (Monmouth Junction, United States), and PAL (Cat. No. D108405, Purity (GC) > 96.5%) was purchased from Sigma-Aldrich Chemical Company (St Louis, United States). These agents were dissolved in saline. The specific dosage used was determined based on cytological experiments in our laboratory under the guidance of modern pharmacological methods. In brief, Uniform and orthogonal design formulas were applied to divide into groups and components, the optimal effective dose of each component and their optimal ratio were determined, and the ratio of DSS, SAL-A, SAL-B and PA was 150:7:300: 500, their concentrations were 1.5 × 10^−4^, 7 × 10^−6^, 3 × 10^−4^, and 5 × 10^−4^ mol/L respectively, and further their effective work dosages were clarified (Shao et al., 2022). The chemical structures of the main components identified in the aqueous extract of *S. miltiorrhiza Bunge* are shown in Figure 1.

### Experimental animals and treatment

Twenty-seven 12-week-old male SHR (250 ± 20 g) and eleven 12-week-old male Wistar-Kyoto (WKY) rats (250 ± 20 g) were purchased from Beijing Vital River Laboratory Animal Technology Co., Ltd (Beijing, China). All animals were housed in a temperature (22°C ± 2°C) and humidity-controlled (40%–70%) animal room with free access to standard chow and tap water. The procedures were approved by the Committee on Animal Care and Use of Hebei University of Chinese Medicine and complied with the Guide for the Care and Use of Laboratory Animals published by the US National Institutes of Health (NIH publication, eighth edition, 2011). Twenty-four SHR were randomly divided into three groups (n = 8 in each group). SHR (SHR model group), perindopril (PD, SHR treated with perindopril, which served as positive control), and SABP (SHR rats treated with SABP). Eight WKY rats served as normal control group.

Perindopril or SABP was administered to SHR as previously reported (Zhang et al., 2016). Briefly, perindopril was injected intraperitoneally daily (0.4 mg/kg/d) in the SHR rats, SABP was injected intraperitoneally daily in the SHR rats, SABP was composed of DSS (5 mg/kg/d), SAL-A (0.233 mg/kg/d), SAL-B (10 mg/kg/d), and PAL (17 mg/kg). SHR and WKY rats were subjected to daily intraperitoneal injection of equal amounts of saline, All animals were given intraperitoneal injection for 8 weeks.

The blood pressure was measured using a tail cuff system (BP-2000R, Visible Systems, United States) as described in the instructions, once a week for eight consecutive weeks, and each time at a certain time (10 a.m.–3 p.m.). At least nine valid values are recorded for each point. Simultaneously. Additional WKY rats were randomly divided into three groups (n = 8) to test the effects of SABP on BP of normotensive WKY rats: perindopril group was injected intraperitoneally daily (0.4 mg/kg/d) in the WKY rats, SABP group was injected intraperitoneally daily (SABP dosage is the same as before) in the WKY rats, WKY group, WKY rats were subjected to daily intraperitoneal injection of equal amounts of saline. The rats in each group were fed with conventional diet, all animals were given intraperitoneal injection for 8 weeks. The same method is used to monitor the BP of WKYs.

### Transmission electron microscopy

Thoracic aortic tissues from three rats in each group were randomly selected, fixed with 2.5% glutaraldehyde fixative (SenBeiJia Biological Technology Co., Ltd., China) for 2 h at room temperature, and washed with 0.1 M of phosphate buffer Saline (PBS pH 7.4) solution containing KH_2_PO_4_ and Na_2_HPO4 (Sigma-Aldrich Chemical Company, St Louis, United States). Then the tissues were post-fixed in 1% osmium tetroxide (Beijing Bellancom Chemistry, China) at room temperature for another 2 h and washed with the phosphate buffer again. The tissues were sequentially dehydrated in 50%, 70% and 90% ethanol, then in a mixture of 45% ethanol and 45% acetone, then in 90% acetone (soak in each solution for 20 min at 4°C). Afterward, the tissues were dehydrated in 100% acetone for 20 min at room temperature and embedded in epoxy resin (Sigma-Aldrich Chemical Company, St Louis, United States). Ultrathin sections (70 nm) were made of the tissue and stained in a solution containing 3% uranyl acetate and lead citrate. Cell morphology and mitochondrial structure were observed with a JEM-1230 transmission electron microscope (JEOL Ltd., Tokyo, Japan), and images were captured by a CCD camera (Olympus, Tokyo, Japan).

### Histochemical staining

Thoracic aorta from the remaining five rats in each group was fixed overnight at 4°C in 10% formalin, then dehydrated in gradient ethanol, embedded in paraffin and sectioned (5 μm). After dewaxing and hydration, the remodeling of the vascular tissues was detected by hematoxylin-eosin (HE) staining kit (Beijing Solabio Life Sciences Co., Ltd., China) as per the manufacturer’s protocol. The sections were subjected to antigen repair and sealing, followed by immunohistochemical staining to measure the expression of relevant genes. ImageJ software was used to measure the optical density (OD) values of gene expression. The antibodies used for immunohistochemical staining are listed Table 1.

**TABLE 1:** List of antibodies for immunohistochemical staining.

Reagent or resource	Source	Identifier	Ihc	Wb
Antibodies				
Rabbit monoclonal anti-Nox1	Biorbyt	orb6469	√	√
Rabbit monoclonal anti-Nox2	Biorbyt	orb1105610	√	√
Rabbit monoclonal anti-Nox4	Biorbyt	orb97102	√	√
Rabbit monoclonal anti-ROS	Biorbyt	orb13678	√	
Rabbit monoclonal anti-GAPDH	Cell Signaling Technology	mAb#5174		√
Rabbit monoclonal anti-TLR4	Cell Signaling Technology	mAb#14358		√
Rabbit monoclonal anti-NF-KB	Cell Signaling Technology	mAb#8242	√	√
Rabbit monoclonal anti-IL-6	Cell Signaling Technology	mAb#12912		√
Rabbit monoclonalanti-Smad3	Cell Signaling Technology	mAb#9523		√
Rabbit monoclonal anti-TGF-P1	Cell Signaling Technology	mAb#70667		√
Rabbit monoclonal anti-p-Smad3	Cell Signaling Technology	mAb#9520		√
Rabbit monoclonal anti-p-NF-KB	Cell Signaling Technology	mAb#3033		√
Rabbit monoclonal anti-a-SMA	Abeam	ab7817	√	√
Rabbit monoclonal anti-Collagen	Abeam	ab138492	√	√
Rabbit monoclonal anti-IL-6	Abeam	ab6992	√	
Rabbit monoclonalanti-CTGF	Abeam	ab6992	√	
Rabbit monoclonal anti-TGF-P 1	Abeam	ab215715	√	

### Western blot

Cells or tissues were lysed using radio immunoprecipitation assay lysis buffer (Beyotime, Beijing, China) supplemented with protease inhibitors. The protein concentration of the dissolved product was measured using the bicinchoninic acid assay Protein Assay Kit (Thermo Fisher Scientific Inc., Waltham, United States). After separation by 10% SDS-PAGE, the proteins were transferred to polyvinylidene difluoride membranes. The membranes were then incubated overnight at 4°C with the primary antibodies (Table 1) and then with horseradish peroxidase (HRP)-conjugated secondary antibodies for another 1.5 h at room temperature. The ECL kit (Abcam, Cambridge, United Kingdom) was used to visualize the protein bands, and Quantity One imaging J software (Bio-Rad Laboratories, Hercules, United States) was used to quantify the proteins with GAPDH as an internal reference.

### Isolation and culture of adventitial fibroblasts

Vascular adventitial fibroblasts (AFs)were primarily cultured from the thoracic aorta of SHR and WKY rats according to a previous report (An et al., 2015). Briefly, after euthanasia of rats by an overdose of sodium pentobarbital (200 mg/kg), the thoracic aorta was removed and cleaned under aseptic conditions. The adventitia was cut into 1–2 mm^2^ and grown on a culture dish with Dulbecco’s modified Eagle’s/F12 medium (Invitrogen Inc., Carlsbad, United States) supplemented with 20% fetal bovine serum (Gibco, Carlsbad, United States) and 1% penicillin/streptomycin solution (Sigma-Aldrich Chemical Company, St Louis, United States) in 5% CO_2_ at 37°C. The culture medium was refreshed every 48 h. Cells grown to confluence were subsequently collected and passaged with 0.25% trypsin solution (Sigma-Aldrich Chemical Company, St. Louis, United States). The cells at passage three to five were selected for the following experiments.

The effective action time and concentration of SABP were analyzed by conducting concentration course and time course of SABP effects at the cell level. The cells were treated with 0.2×SABP working solution containing DSS (1.5 × 10^−4^ mol/L), SA-A (7 × 10^−6^ mol/L), SA-B (3 × 10^−4^ mol/L) and PAL (5 × 10^−4^ mol/L) (Hao et al., 2017). AFs treated with 100 μmol PD solution served as a positive control.

### Cell counting kit-8 (CCK-8)

Cell proliferation was examined by the CCK-8 assay. Briefly, the cells were seeded into 96-well plates at a density of 1×10^3^ cells/well. After the relevant treatment, 10 μL CCK-8 (Dojindo Laboratories, Kumamoto, Japan) was supplemented to each well, and incubation continued for 4 h. The proliferative activity of the cells was measured by reading the optical density (OD) value at 450 nm with the help of a microplate reader (Thermo Fisher Scientific Inc., Waltham, United States).

### Cell wound healing assay

The cells were seeded into six-well plates at 1×10^5^ cells/well. A 200 μL sterile pipette tip was utilized to make a fine scratch along the center of each well with the same width. Next, the cells were incubated in serum-free medium containing 0.2×SABP mmol/L for 0 and 24 h, the 24 h migration of cells was measured by ImageJ software.

### Data analysis

Results were expressed as means ± SEM, and all statistical analyses were performed using SPSS 22.0 (IBM Corp. Armonk, United States). Each observation was reproduced in the cells isolated from at least three different animals. Differences among multiple groups were analyzed by one-way or two-way analysis of variance (ANOVA), followed by Tukey’s *post hoc* test. A value of *p* < 0.05 was considered statistically significant.

## Results

### SABP lowed blood pressure and recovered ultrastructure changes of AFs in thoracic aorta

**FIGURE 2:**
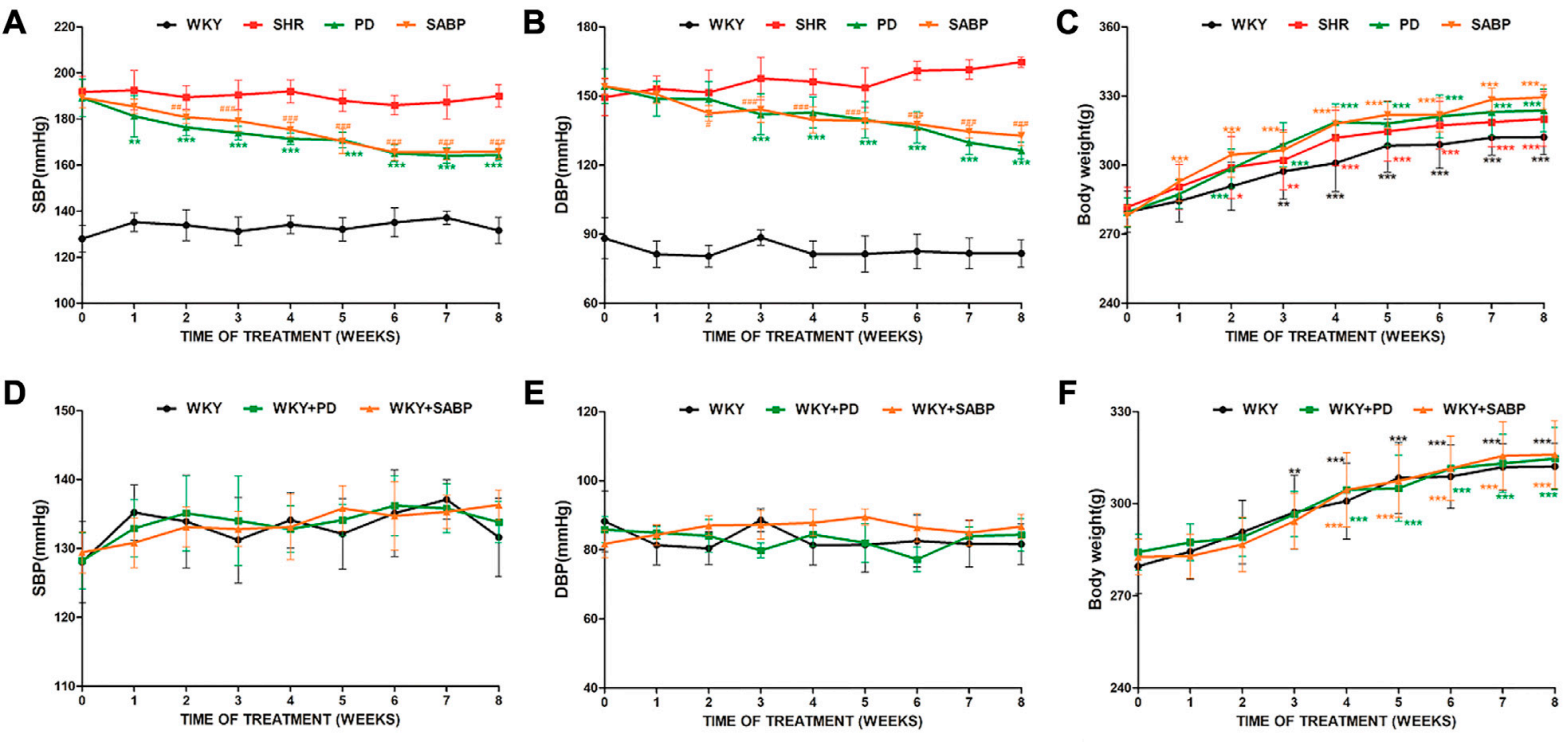
Effect of SABP on blood pressure and body weight in SHR and WKY. The systolic blood pressure**(A)**, diastolic blood pressure **(B)** and body weight **(C)** in SHR after SABP or perindopril administration (n = 8, *p < 0.05, **p < 0.01, ***p < 0.001 vs. SHR; #*p* < 0.05, ##*p* < 0.01, ###*p* < 0.001 vs. SHR). The systolic blood pressure **(D)**, diastolic blood pressure **(E)** and body weight **(F)** in the normotensive WKY rats after SABP or perindopril administration. There was no significant change in systolic blood pressure or diastolic blood pressure within and between groups, and there was no significant difference in body weight between groups. However, there was a significant increase in body weight from the third week of comparison within the group (n = 8, *p < 0.05, **p < 0.01, ***p < 0.001). WKY and SHR are treated with perindopril and SABP by intraperitoneal injection daily.

First, we assessed the effects of SABP and perindopril on the blood pressure, inflammation, oxidative stress, fibrosis, and vascular remodeling in SHRs and the WKYs with normal blood pressure. The administration of SABP or perindopril was performed *via* intraperitoneal injection daily for 8 weeks. The systolic blood pressure in SHRs was significantly higher than WKY rats. Either SABP or perindopril significantly reduced the systolic blood pressure in SHR starting at the first week of the treatment and became apparent from day 7. Treatment for 6 weeks decreased the systolic blood pressure and diastolic blood pressure of SHR by 24.9 ± 8.53 and 21.5 ± 6.33 mmHg, respectively, compared with the baseline values. There was no significant difference in the reduction of systolic blood pressure between the SABP and perindopril treatment, suggesting both drugs had comparable effects on systolic blood pressure (Figures 2A, B). In WKY rats, SABP or perindopril treatment did not significantly change systolic blood pressure or diastolic blood pressure (Figures 2D, E). There was a significant difference in body weight from the third week within the groups (Figures 2C–F).

**FIGURE 3:**
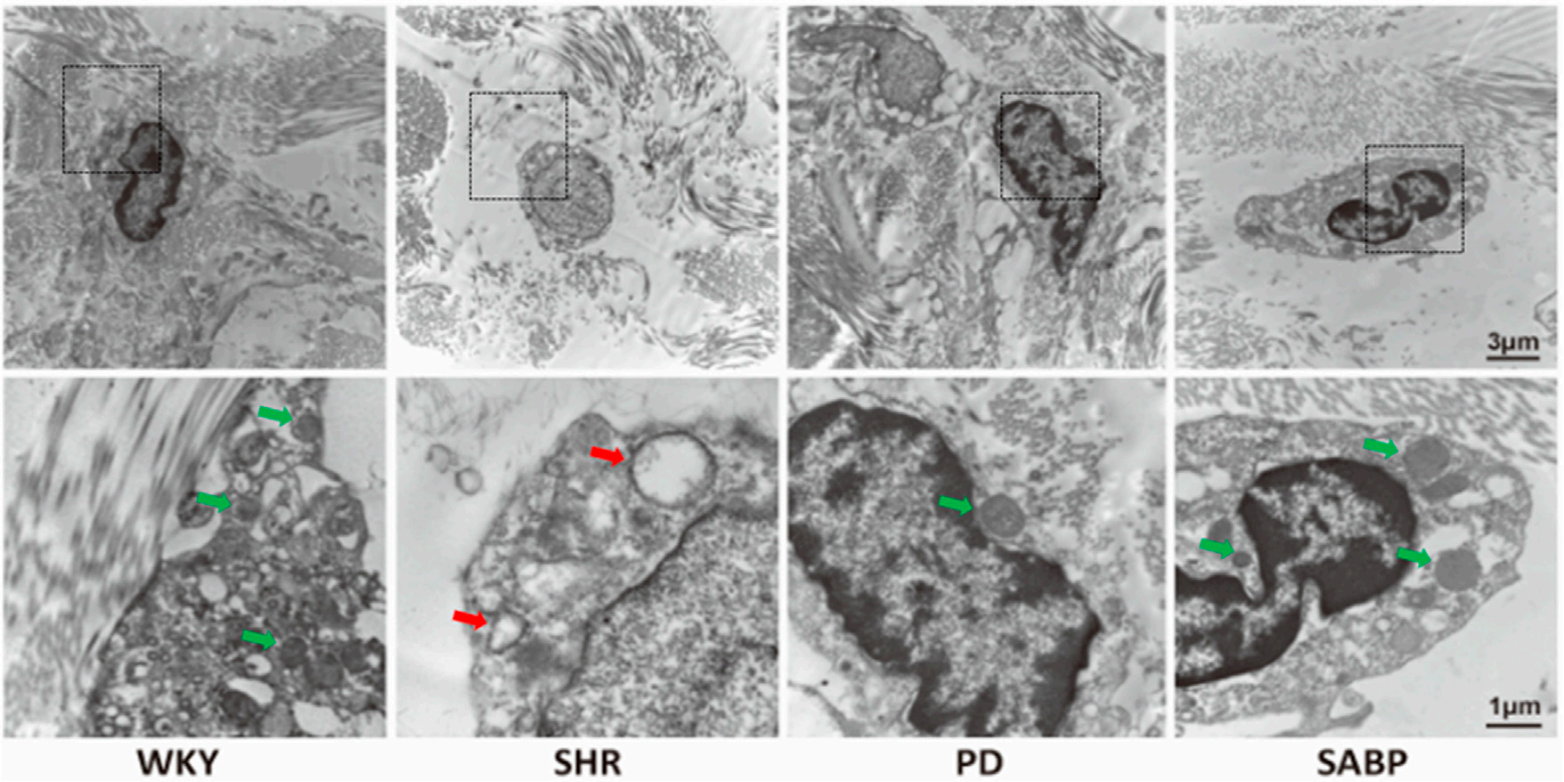
Effect of SABP on the ultrastructure of AFs of thoracic aorta in SHR. Ultrastructure of mitochondria in AFs of thoracic aorta in SHR and WKY observed by transmission electron microscopy (n = 3) (the red arrow represents the mitochondria in the pathological state, and the green arrow represents the normal mitochondrial structure and recovery to normal state). WKY and SHR are treated with perindopril and SABP by intraperitoneal injection daily.

The ultrastructure of thoracic aorta was assessed by transmission electron microscopy. The mitochondria in the AFs of the thoracic aorta of SHR were swollen, with broken or absent mitochondrial cristae and vacuolization, while in the WKY group, the mitochondria were normal morphology with intact mitochondrial structures (Figure 3). Treatment with SABP or perindopril reduced the breakage of mitochondrial cristae in the thoracic aorta of SHRs.

### SABP ameliorates inflammation in the thoracic aorta of SHRs

**FIGURE 4:**
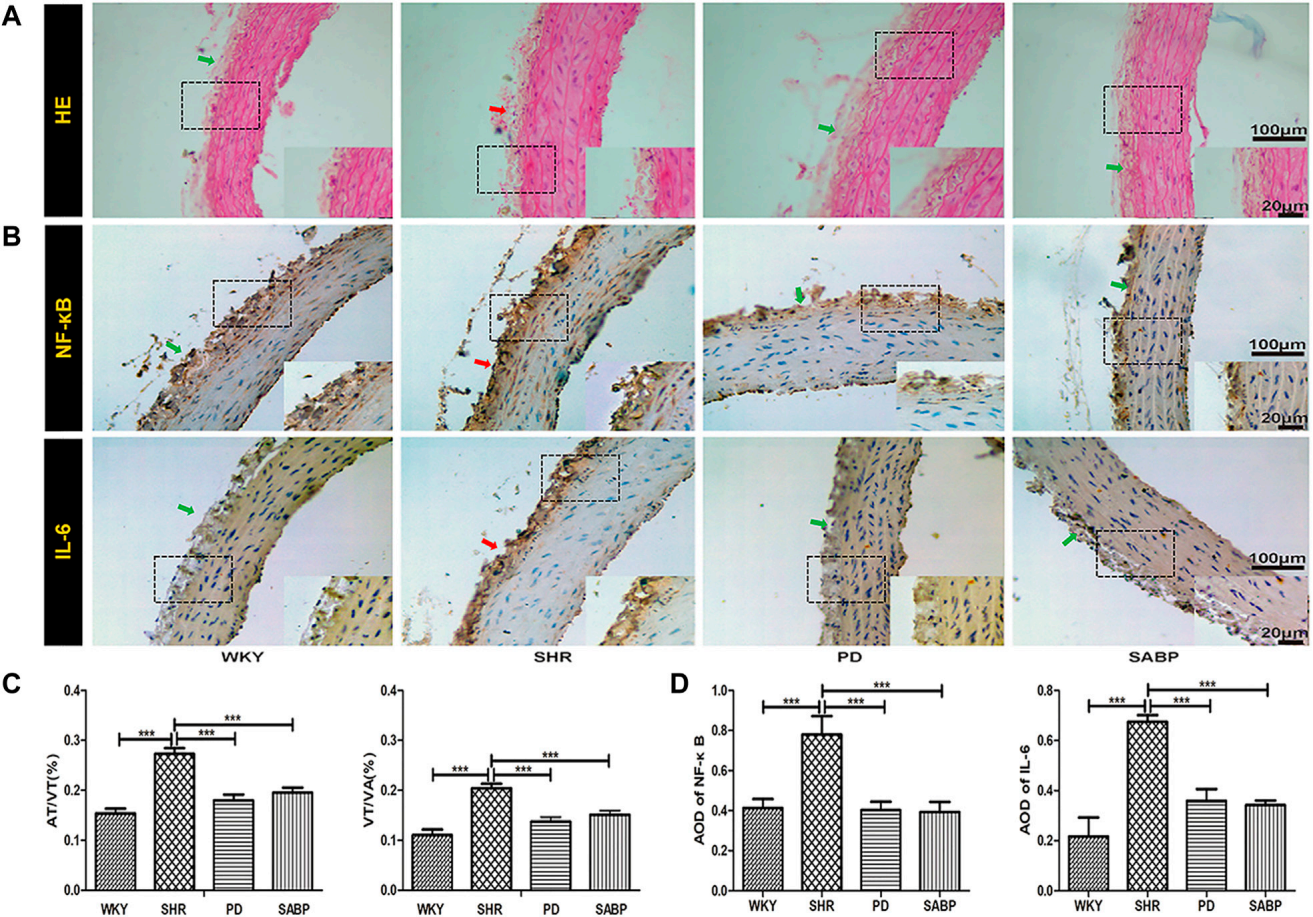
Effect of SABP on vascular pathology and inflammatory response in the adventitia of thoracic aorta of SHR. **(A)** The H&E stain of the aorta in rats were imaged with optical microscopy at ×200 magnification. **(B)** Representative figure of immunohistochemical (IHC) staining of the expression of inflammation-related indicators in adventitia of aortic cross-section (n = 5). The red arrow indicates the adventitia of the thoracic aorta in the model SHR, the green arrow indicates the adventitia of the thoracic aorta in the WKY group and in the SABP and perindopril intervention groups. **(C)** The aorta thickness was analyzed by ImageJ software. Measured in millimeters as the standard unit, the thickness of five aorta (mm) in each slice was measured and the percentage of the adventitia thickness/the vessel wall thickness (AT/VT) and the vessel wall thickness/radius (VT/VA) of the thoracic aorta in all groups was calculated separately (n = 5, ***p < 0.001 vs. SHR). **(D)** The average optical density value of pNF-κB and IL-6 by IHC Staining on adventitia in the five experimental groups (Mean ± SEM, n = 5). ***p < 0.001 vs SHR. WKY and SHR are treated with perindopril and SABP by intraperitoneal injection daily.

Histopathological analysis showed that the adventitia and vascular wall of thoracic aorta were thickened in SHRs compared with those in WKY rats, with hypertrophy and proliferation of vascular smooth muscle cells, and cell maladjustment observed. SABP or perindopril treatment reduced the thickening of the adventitia and thoracic aorta vessel wall and prevent morphological changes of vascular smooth muscle cells. The ratio of adventitia/vessel wall thickness (AT/VT) and ratio of vessel wall/radius (VT/VA) thickness were significantly lower than those in the SHRs, and their morphological manifestations were similar to those in WKY rats (Figures 4A–C). The expression of inflammation-related mediators, NF-κB and IL-6, was significantly reduced after treatment with SAMP or perindopril (Figures 4B–D).

### SABP ameliorates oxidative stress in the thoracic aorta of SHR

**FIGURE 5:**
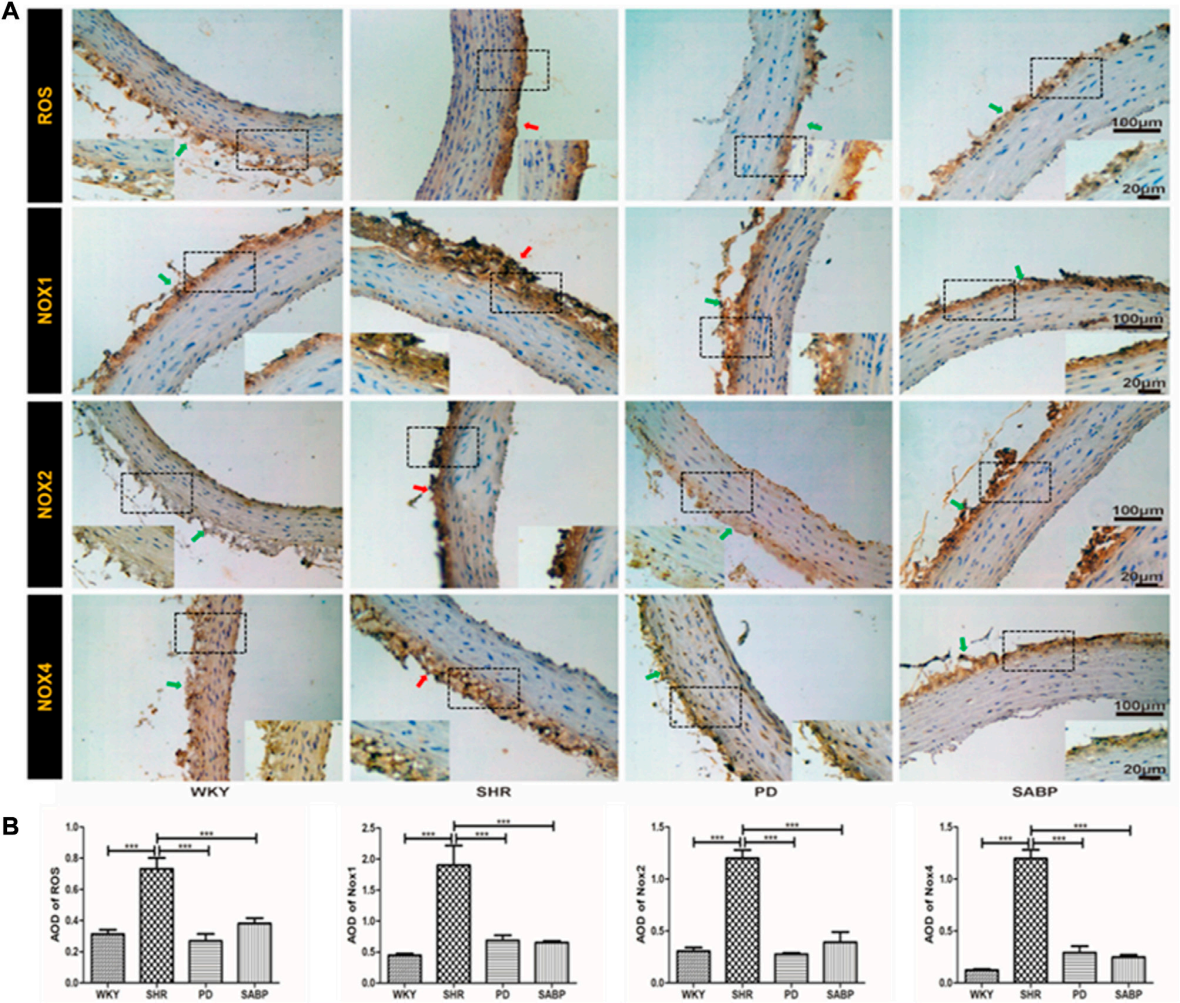
Effect of SABP on oxidative stress in the adventitia of thoracic aorta of SHR. **(A)** Representative figure of IHC staining of the expression of oxidative stress-associated indicators (n = 5). The red arrow indicates the adventitia of the thoracic aorta in the model SHR, the green arrow indicates the adventitia of the thoracic aorta in the WKY group and in the SABP and perindopril intervention groups. **(B)** The average optical density value of ROS, NOX1, NOX2 and NOX4 by IHC Staining on adventitia in the five experimental groups (Mean ± SEM, n = 5). ***p < 0.001 vs SHR. WKY and SHR are treated with perindopril and SABP by intraperitoneal injection daily.

Oxidative stress-related indicators in the thoracic aorta of each group were detected by immunohistochemistry (Figure 5A), followed by a quantification based on the optical density values (Figure 5B). We found that ROS, NOX1, NOX2, and NOX4 levels were significantly higher in adventitia in the SHR than in WKY rats. In contrast, these indicators were significantly reduced in SHR treated by SABP or perindopril to the levels in WKY (Figure 5).

### SABP ameliorated fibrosis in the thoracic aorta of SHRs

**FIGURE 6:**
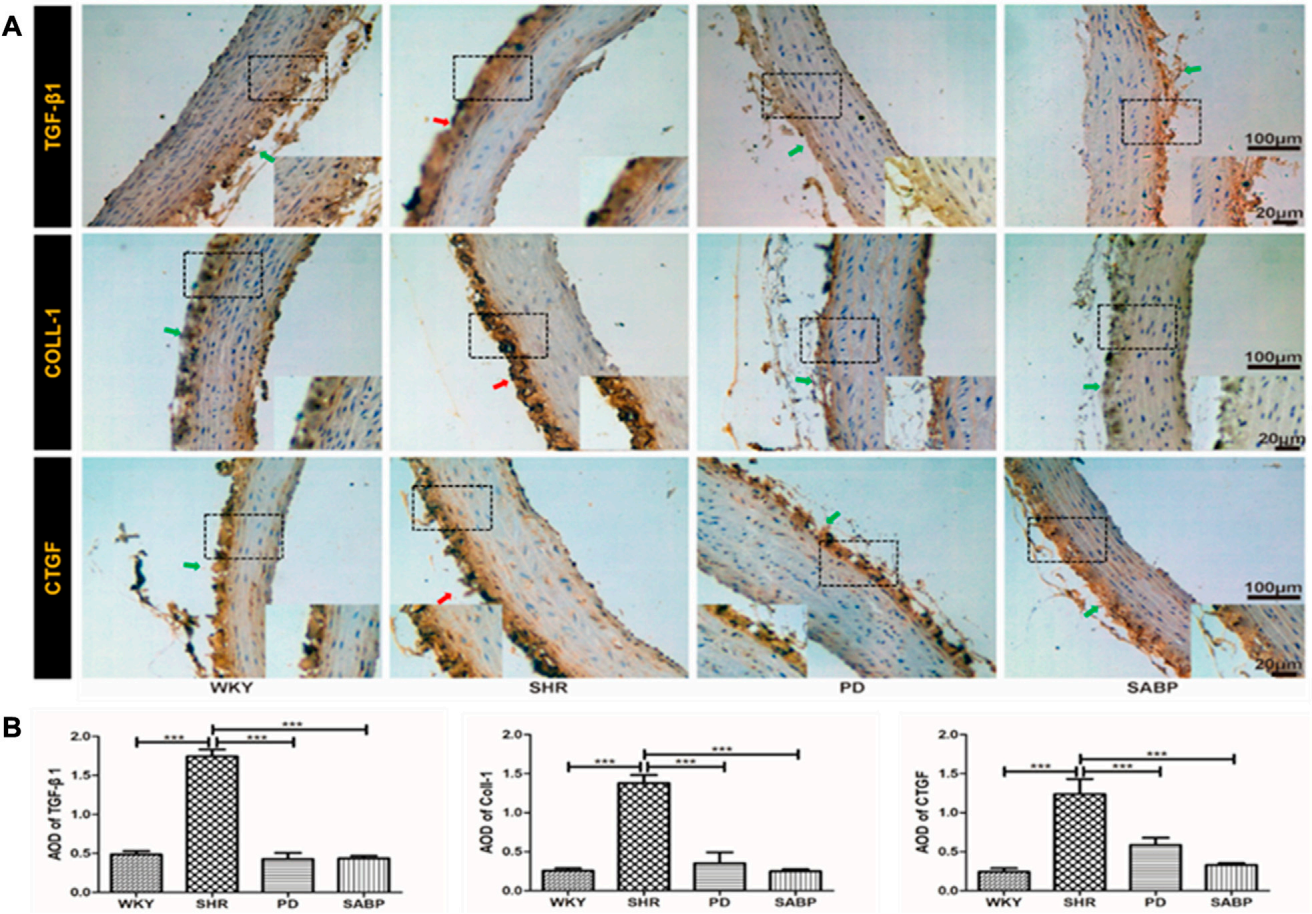
Effect of SABP on fibrosis in the adventitia of thoracic aorta of SHR. **(A)** Representative figure of IHC staining of the expression of fibrosis-related indicators in adventitia of aortic cross-section. (n = 5). The red arrow indicates the adventitia of the thoracic aorta in the model SHR, the green arrow indicates the adventitia of the thoracic aorta in the WKY group and in the SABP and perindopril intervention groups. **(B)** The average optical density value of TGF-β, COLL1 and CTGF by IHC Staining on adventitia in the five experimental groups (Mean ± SEM, n = 5). ***p < 0.001 vs SHR. WKY and SHR are treated with perindopril and SABP by intraperitoneal injection daily.

The expression of fibrosis indicators, TGF-β1, COL-1 and CTGF, in the thoracic aorta of each group was detected by immunohistochemical staining and then the optical density was quantified (Figures 6A, B). A significant increase in COL-1, CTGF and TGF-β1 expression was found in the SHRs compared with WKY rats, while perindopril or SABP treatment significantly suppressed the expression of fibrosis-related indicators in SHRs.

### SABP ameliorated inflammation, oxidative stress and vascular remodeling in the thoracic aorta and kidneys in SHRs

**FIGURE 7:**
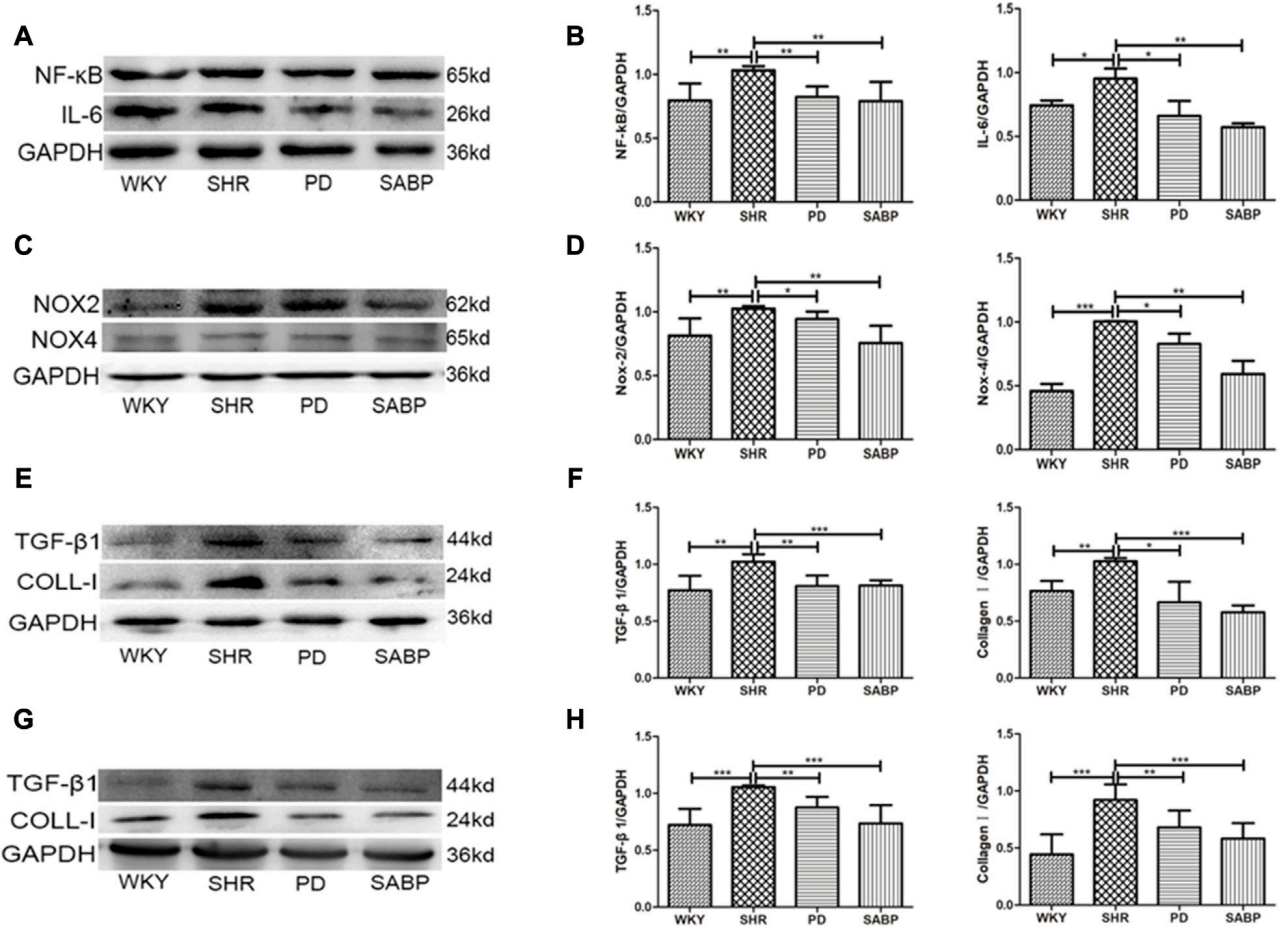
Effect of SABP on inflammatory response, oxidative stress and fibrosis in the adventitia of thoracic aorta of SHR. **(A)** Representative figure of WB of the expression of inflammation-related proteins (NF-kB and IL-6) in adventitia of aorta (n = 5). **(B)** The average optical density value of NF-kB and IL-6 by WB in the five experimental groups (Mean ± SEM, n = 5). ***p < 0.001 vs. SHR. **(C)** Representative figure of WB of the expression of oxidative stress-related proteins (NOX2 and NOX4) in adventitia of aorta (n = 5). **(D)** The average optical density value of NOX2 and NOX4 by WB in the five experimental groups (Mean ± SEM, n = 5). ***p < 0.001 vs. SHR. **(E)** Representative figure of WB of the expression of fibrosis-related proteins (TGF-βand COLL-1) in adventitia of aorta (n = 5). **(F)** The average optical density value of TGF-βand COLL-1 by WB in the five experimental groups (Mean ± SEM, n = 5). ***p < 0.001 vs. SHR. **(G)** Representative figure of WB of the expression of fibrosis-related proteins (TGF-βand COLL-1) in the kideny (n = 5). **(H)** The average optical density value of TGF-β and COLL-1 by WB in the three experimental groups (Mean ± SEM, n = 3). ***p < 0.001 vs. SHR. WKY and SHR are treated with perindopril and SABP by intraperitoneal injection daily.

Using Western blot analysis, we found that the expression levels of NF-κB and IL-6 were significantly elevated in the thoracic aorta of SHRs, and that SABP or perindopril treatment drastically reduced the increases in expression of these pro-inflammatory factors (Figures 7A, B). Additionally, the protein levels of NOX2 and NOX4 were significantly increased in the thoracic aorta of SHRs while SABP or perindopril treatment significantly reduced the elevation of NOX2 and NOX4 levels in SHRs (Figures 7C, D). Furthermore, the expression of fibrosis-related proteins TGF-β1 and COL-1 in the thoracic aorta of each group of rats was detected. TGF-β1 and COL-1 levels were significantly elevated in SHR, while SABP and perindopril treatment mitigated the elevation of these proteins in SHRs (Figures 7E, F). In addition, the expression of TGF-β1 and COL-1 was also significantly augmented in the kidney tissues of SHRs, and SABP or perindopril treatment reduced the expression of these proteins (Figures 7G, H). The tissue samples used for Western blot analysis were whole blood vessels and whole kidney tissue. These findings suggest that SABP treatment ameliorates the inflammatory responses, oxidative stress in thoracic aorta and ameliorates the fibrosis in thoracic aorta and kidney in SHRs.

### SABP inhibited the proliferation, migration and differentiation of AFs in SHRs

**FIGURE 8:**
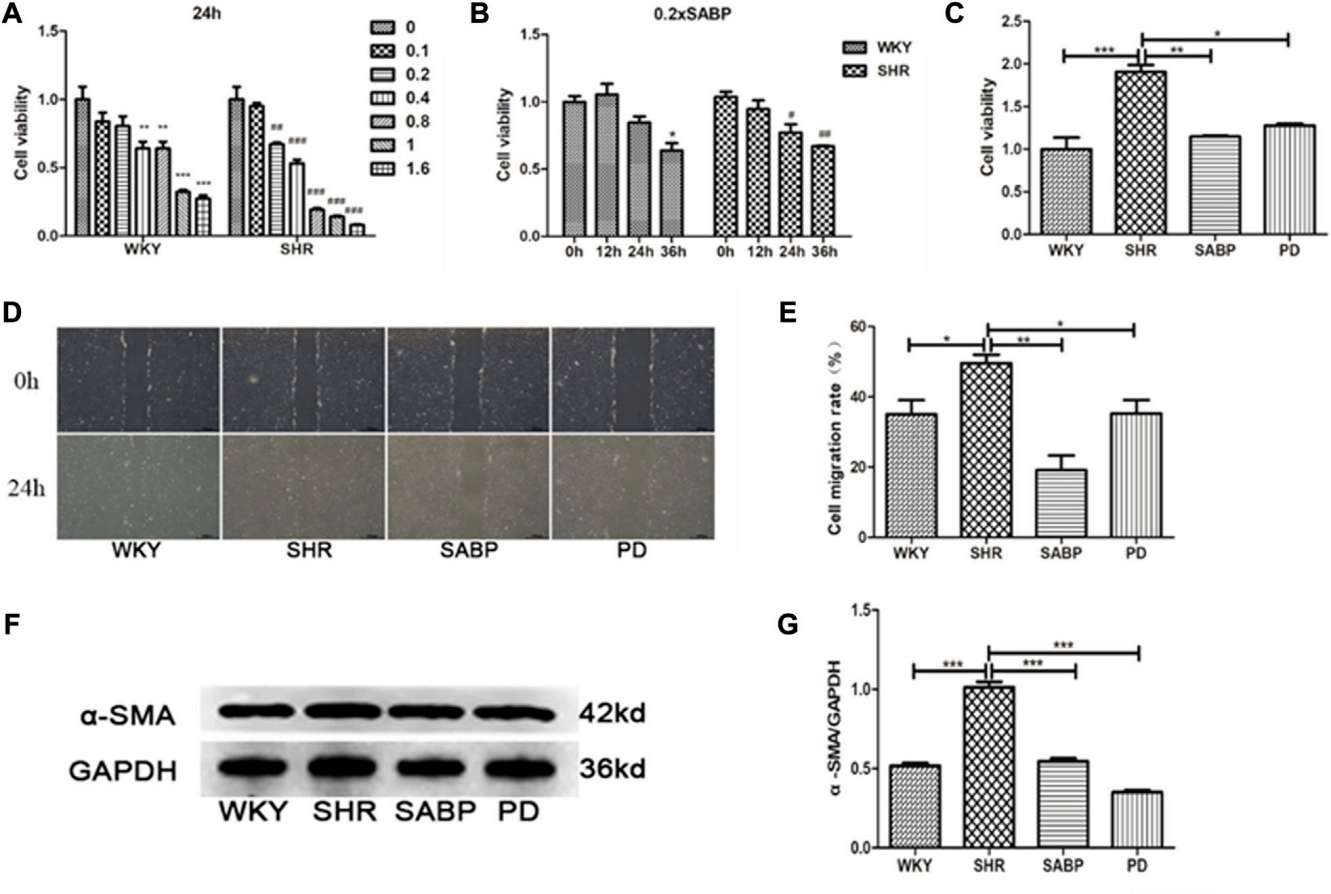
Effect of SABP on proliferation, migration and differentiation of adventitial fibroblasts (AFs) from SHR. **(A)** Concentration-dependent effect of SABP on AFs proliferation in 24 h, SABP concentrations (0–1.6×SABP mmol/L) were added to AFs for 24 h and **(B)** the time-dependent effect of 0.2×SABP on AFs proliferation, SABP (0.2×) was added to AFs for a continuous increase of time (0–36 h), and then the cell viability was observed by CCK8. **(C)** The effect of 0.2×SABP on AFs proliferation of in 24 h, 0.2×SABP was added to AFs for 24 h and it was observed that SABP inhibited the proliferation of AFs compared with SHR. **(D)** Representative of AFs migration capability in AFs was observed by cell wound healing assay. **(E)** The effect of SABP on AFs migration capability was evaluated, SABP inhibited the migration of AFs was observed. **(F)** Representative of α-SMA expression in AFs was observed by Western blot. **(G)** The effect of SABP on α-SMA expression in AFs was evaluated, SABP inhibited α-SMA expression in AFs was observed. Each image is a representative example of the three experiments. Values are expressed as means ± SEM (*p < 0.05, **p < 0.01, ***p < 0.001). WKY and SHR are treated with perindopril and SABP by intraperitoneal injection daily.

Using isolated and cultured AFs from the thoracic aorta of SHRs and WKY rats, we found that SABP inhibited the proliferation of AFs in a concentration- and time-dependent manner (Figures 8A, B). Through detecting the cell viability using CCK8 assay, SABP treatment inhibited the proliferation of AFs obtained from SHRs compared with untreated group (SHRs). The perindopril was used as a positive control group (Figure 8C). Cell migratory capacity was then examined by wound healing assay. We found that the migratory capacity of AFs in SHRs was significantly elevated, whereas SABP or perindopril significantly inhibited the migratory capacity of AFs (Figure 8D). The expression of α-SMA, a marker of AFs to myofibroblast transformation, was detected by Western blot. We found that α-SMA expression was significantly increased in AFs extracted from SHR, while SABP or perindopril treatment inhibited the elevated α-SMA expression in SHRs (Figure 8E).

### SABP inhibited oxidative stress, inflammation, and fibrosis in AFs of SHRs

**FIGURE 9:**
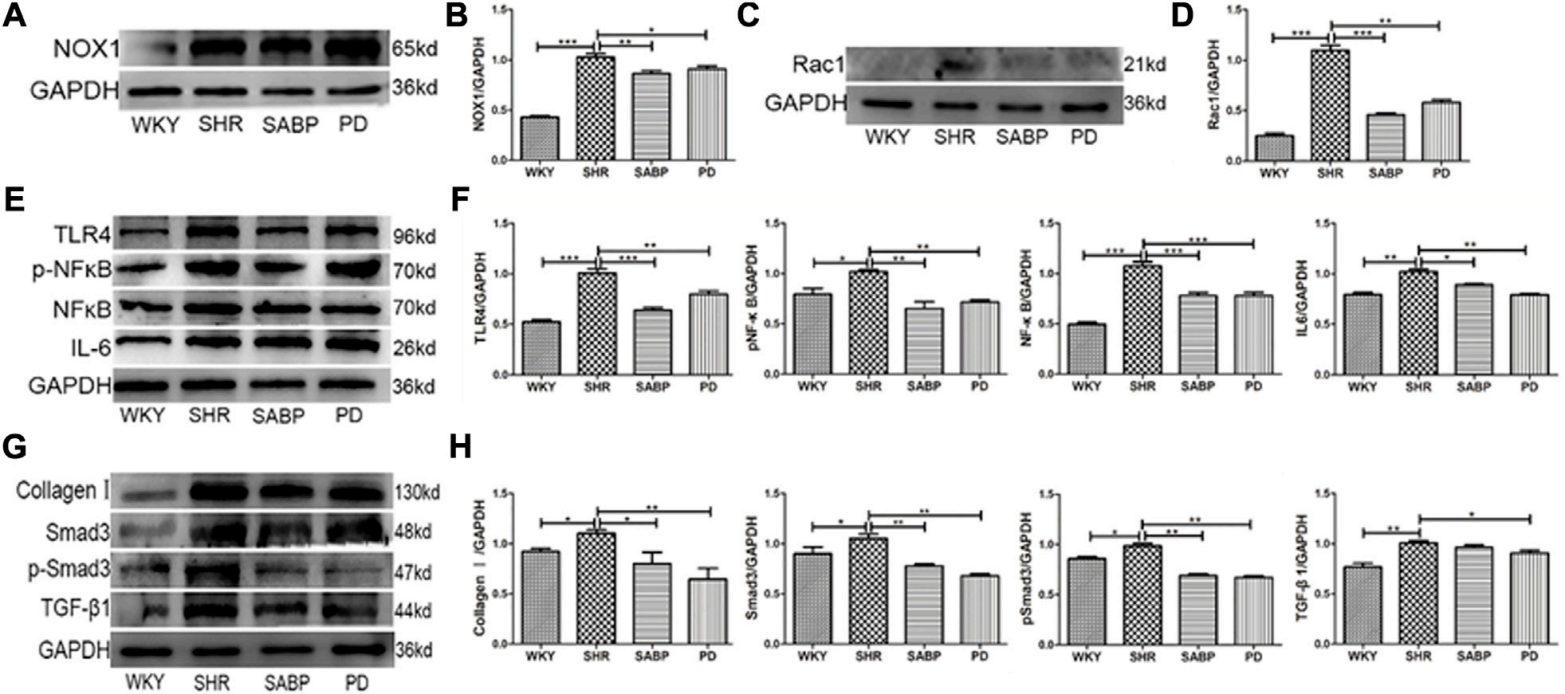
Effects of SABP on oxidative stress, inflammation, and fibrosis in AFs. **(A)** Representative of NOX1 expression in AFs examined by Western blot. **(B)** The effect of SABP on NOX1 expression was evaluated and SABP inhibited NOX1 expression was observed. **(C)** Representative of Rac1(mitochondrial damage marker protein) expression in AFs examined by Western blot. **(D)** The effect of SABP on Rac1 expression was evaluated and SABP inhibited Rac1 expression was observed. **(E)** The expression of inflammatory factors TLR4, NF-κB, pNF-κB and IL-6 in AFs examined by Western blot. **(F)** The effect of SABP on TLR4, NF-κB, pNF-κB and IL-6 was evaluated and SABP inhibited expression of TLR4, NF-κB, pNF-κB and IL-6 was observed. **(G)** The expression of fibrosis-associated proteins TGF-β, Smad3, pSmad3 and COL-1 in AF examined by Western blot. **(H)** The effect of SABP on TGF-β, Smad3, pSmad3 and COL-1 was evaluated and SABP inhibited expression of TGF-β, Smad3, pSmad3 and COL-1 was observed. Each photograph is a representative example of the three experiments. Values are expressed as means ± SEM (*p < 0.05, **p < 0.01, ***p < 0.001 vs*.* SHR). WKY and SHR are treated with perindopril and SABP by intraperitoneal injection daily.

We finally examined the effect of SABP on the expression of NOX1 using Western blot in AFs (Figure 9A). NOX1 protein levels in AFs were increased significantly in SHR, but were inhibited by SABP or perindopril treatment (Figure 9B). Also, we found that the expression level of Rac1, a mitochondrial damage marker protein, was significantly elevated in Afs of SHRs, whereas SABP or perindopril treatment suppressed the elevated Rac1 levels in SHRs (Figures 9C, D). The expression levels of TLR4, NF-κB, pNF-κB and IL-6 were significantly increased in the AFs of SHRs. SABP or perindopril treatment inhibited the expression of these pro-inflammatory factors. Finally, we also measured the expression levels of fibrosis-related proteins in AFs of SHRs (Figures 9E, F), and found that the expression levels of TGF-β1, Smad3, pSmad3 and COL-1 were significantly elevated in AFs of SHR, and SABP or perindopril treatment inhibited the expression of these proteins to suppress the fibrosis (Figures 9G, H).

## Discussion

Hypertension, a major risk factor for cardiovascular and cerebrovascular events (Xiong et al., 2013), negatively impacts one’s quality of life. However, current treatments for hypertension are limited by the availability, cost, and adverse effects of anti-hypertensive drugs. As a result, complementary and alternative medicine, including traditional Chinese medicine, is becoming a popular alternative strategy (Xiong et al., 2015). Our study found that SABP, the active metabolites of *S. miltiorrhiza Bunge*, has a potent anti-hypertensive effect that is comparable to the effect of perindopril-induced depressor effect in SHRs. In addition, we found that SABP treatment effectively alleviated oxidative stress, inflammation as well as fibrosis in the thoracic aorta and kidneys in SHRs. This antihypertensive effect is partially due to the inhibition of oxidative stress, inflammatory response and fibrosis in AFs.

Early adventitia remodeling is a fundamental finding in experimental models of systemic vascular injury and hypertension (Han et al., 2018; Chen et al., 2021). Hypertension, atherosclerosis and vascular injury have been linked to early adventitial fibroblast proliferation and monocyte/macrophage accumulation (Siow et al., 2003; Wang et al., 2021a). In a model of chronic nitric oxide inhibition, which leads to systemic hypertension, the thickness of adventitia and the number of cells increased before change in the media or intima (Schulze-Bauer et al., 2002). Under conditions of elevated blood pressure, the adventitia becomes the dominant component of the vascular wall due to its obvious stiffening (Liu et al., 2021). As a result, adventitial fibroblasts are considered to be the most sensitive cells to detect hypertensive states (McGrath et al., 2005). In hypertension, increased vascular ROS production through NADPH oxidase, which activates fibroblast response to injury. This, in turn, release a variety of mediators, including ET-1, PDGF, FGF-2, and cyclophilin, which affect vascular tension (Adam et al., 2012; Zhang et al., 2022). Activated adventitial fibroblasts therefore play a crucial role in influencing the tension and structure of the vascular wall after a variety of injuries or stresses. They do so both directly by secreting vasoactive and growth promoting molecules, and indirectly by producing chemokines that promote the accumulation of leukocytes and progenitor cells. Fibroblast are the key drivers of many reactions involved in vascular remodeling. MiR-21 was found to be expressed in fibroblasts (Wang et al., 2014) and it regulates fibroblast fibrosis by upregulating MAPK phosphorylation. In addition, histone deacetylase inhibition of miR-124 in the vascular adventitia can restore the expression of miR-124 and inhibit fibroblast proliferation, suggesting that histone deacetylase inhibitors have potential therapeutic potential (Lassègue et al., 2012; Zhang et al., 2017). These findings support the adventitia as a key regulator of the function and structure of the vascular wall and provide a theoretical foundation for the adventitia as a target for the treatment of hypertension.

Nox enzymes are heteroprotein complexes found in AFs with unique regulatory mechanisms that control various functions in cells, including cell survival, growth and death, differentiation, angiogenesis, and contraction (Drummond et al., 2011). The NOX1 and NOX2 oxidases are the primary sources producing ROS in the artery wall in hypertension and contribute to oxidative stress and vascular inflammation that trigger arterial remodeling (Li et al., 2016). In the present study, we found that the optimal combination of four hydrophilic active metabolites of Salvia miltiorrhiza Bunge, SABP, significantly reduced the levels of NOX1, NOX2, NOX4 and ROS level in the adventitia of thoracic aorta in SHRs and cultured adventitial fibroblasts of thoracic aorta from SHRs. This suggest that SABP may alleviate oxidative stress in hypertension. The elevation of oxidative stress caused by isoproterenol was also suppressed by the combination Paeonol and DSS (Harrison et al., 2011). ROS activates pro-inflammatory transcription factors such as NF-κB, which in turn, modulates the expression of genes encoding adhesion molecules and chemokines, leading to the accumulation of inflammatory cells (Wei et al., 2013). Therefore, we measured pro-inflammatory factors including NF-κB, IL-6 and TLR4 in adventitia of the thoracic aorta in SHRs using both immunohistochemistry and Western blot. We found that there was obvious inflammation in the adventitia of thoracic aorta in SHRs, which was mitigated by SABP treatment. Interestingly, the levels of IL-6 and the nuclear translocation of NF-κB were both evidently decreased by PAL, one of the most active metabolites of SABP in myocardial ischemia/reperfusion injury (Wang et al., 2021b). The protective role of Sal-A has been reported in traumatic brain injury *via* suppression of inflammation and recovery of mitochondrial function (Wan et al., 2019), consistent with our findings.

Fibrosis also leads to cardiovascular remodeling characterized by abnormal collagen architecture. PAL significantly suppresses fibrosis caused by collagen deposition in myocardial tissues (Mennuni et al., 2014). In this study, we found that SABP effectively suppressed the expression of COL-1, CTGF, TGF-β1, and Smad3 in the adventitia of thoracic aorta in SHRs. Renal damage represents a frequent event during hypertension (Huang et al., 2020). In this study, we notice a significant renal fibrosis in SHRs and SABP exhibited strong anti-fibrotic effects on the thoracic aorta and the kidneys. Our data showed that SABP significantly decreased the plasma level of Ang II in SHR, inhibited the expression of ET-1, TGF-β1, Col-I, α-SMA in thoracic aorta, and prevented vascular remodeling (Zhang et al., 2016). However, the role of SABP in the adventitia of the thoracic aorta and kidney fibrosis has rarely been reported before.

AFs can migrate from the adventitia to the media through the external elastic plate, contributing to vascular remodeling (Tieu et al., 2011). In addition, adventitial remodeling featuring the differentiation of AFs into myofibroblasts plays an important role in vascular remodeling in hypertension (An et al., 2015; Barman and Fulton, 2017). Hence, we isolated primary AFs from SHRs and WKY rats to test the effect of SABP on AFs. Our results revealed that SABP prevented the proliferation, migration and differentiation of AFs. It also repressed the expression of factors related to oxidative stress (NOX1 and Rac1), inflammation (TLR4, NF-κB, pNF-κB, and IL-6) as well as fibrosis (TGF-β, Smad3, pSmad3, and COL-1). It has been shown that aortic AFs contributes significantly to vascular inflammation by secreting IL-6 and MCP-1 (Nava and Llorens, 2019) and incude NOX4 overexpression, which in turn induces migration and proliferation and expression of matrix-related genes (Lehoux, 2016).

*In vivo* and *in vitro* experimental studies have shown that the adventitia is a dynamic microenvironment that regulates important vascular functions in disease such as intimal hyperplasia and atherosclerosis (Maiellaro and Taylor, 2007). Without blood flow interface, the adventitia has a profound impact on the function of smooth muscle cells, endothelial cells, macrophages and extracellular matrix (Wei et al., 2013; Guo et al., 2018). Oxidative stress and other factors cause vascular adventitia damage and dysfunction, leading to the activation of adventitial fibroblasts, differentiation of myofibroblasts, collagen deposition, decreased elastic fibers, and increased vascular fibrosis (Stenmark et al., 2013). The sebsequent biological process of this regulation including the migration and proliferation of fibroblasts and myofibroblasts, inflammation, immunity, and changes in extracellular matrix that result in vascular remodeling. Our study showed that SABP, the optimal combination of four hydrophilic active metabolites from *S. miltiorrhiza Bunge*, has anti-inflammatory, anti-oxidative, anti-fibrosis, and anti-vascular remodeling effects on thoracic aortic adventitial *in vivo* and adventitial fibroblasts *in vitro*. These findings suggest that the potential of SABP as an antihypertensive agent by inhibiting adventitial fibrosis. The specific dosage used was determined by uniform and orthogonal design formulas, which determines the optimal combination of each component that is needed to generate optimal effects in our previous study. Although Salvia miltiorrhiza Bunge is a well-studied specie, the effects of this combination of its hydrophilic active metabolites (SABP) on reducing oxidative stress, inflammation and fibrosis of adventitia have not been recognized. This study is considered as an early-stage exploratory study due to the complexity of the SABP combination.

The changes of structure and function of thoracic aorta play a crucial role in the pathogenesis of hypertension. Our study also showed that in the hypertensive animal model, the structure of the thoracic aorta has underwent specific pathological changes. However, it is widely believed that peripheral arteries play a more important role in regulating blood pressure than large vessels such as the aorta. Studies have shown that ETA receptor blockers may have therapeutic potential in improving the structure and function of peripheral blood vessels in patients with aortic sclerosis and hypertension (Guo et al., 2018). Therefore, we plan to focus on the changes of peripheral artery structure and function during the onset of hypertension in future research.

## Conclusion

SABP lowered systolic blood pressure in SHRs through antagonizing vascular remodeling and fibrosis and reducing inflammation and oxidative stress in vascular adventitia. This novel information opens up the possibility of developing new strategical for the treatment of hypertension.

## Data Availability

The raw data supporting the conclusions of this article will be made available by the authors, without undue reservation.

## Abbreviations

AFs, Adventitial Fibroblasts; DSS, Danshensu; PAL, Protocatechuic aldehyde; ROS, Reactive Oxygen Species; SABP, DSS, Sal-A, Sal-B, PAL; SHR, Spontaneous hypertensive rat; Sal-A, Salvianolic acid A; Sal-B, Salvianolic acid B; SM, *Salvia Miltiorrhiza Bunge*; WKY Rat, Wistar-Kyoto Rat.
