# Supplementary material for: Aqueous extract of Salvia miltiorrhiza Bunge reduces blood pressure through inhibiting oxidative stress, inflammation and fibrosis of adventitia in primary hypertension
Source: Front Pharmacol. 2023 Feb 28;14:1093669. doi: 10.3389/fphar.2023.1093669 (PMC10011461; doi:10.3389/fphar.2023.1093669)
Supplement: Supplementary file 1 [file DataSheet1.PDF]

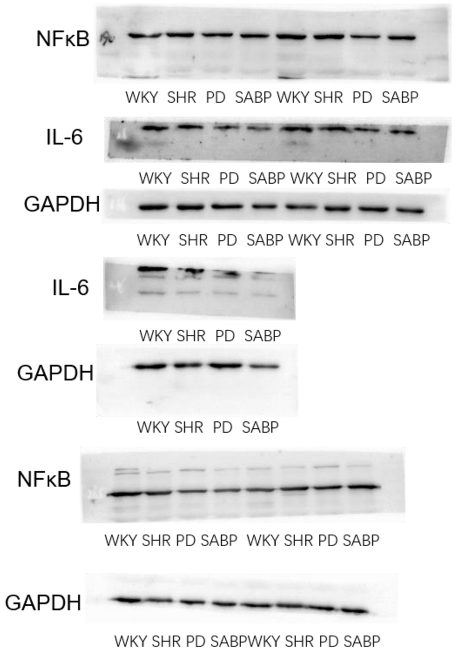

The above Figures are the original western blot of inflammatory detection indicators in the thoracic aorta of SHR in **Figure 6A** of the manuscript, including NF- κ B and IL-6. NAPDH is an internal reference control.

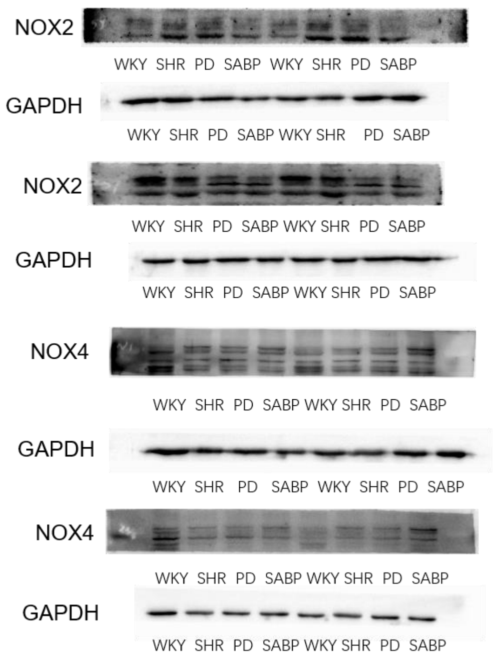

The above figures are the original western blot of oxidative stress detection indicators in the thoracic aorta of SHR in **Figure 6C** of the manuscript, including NOX2 and NOX4. NAPDH is an internal reference control.

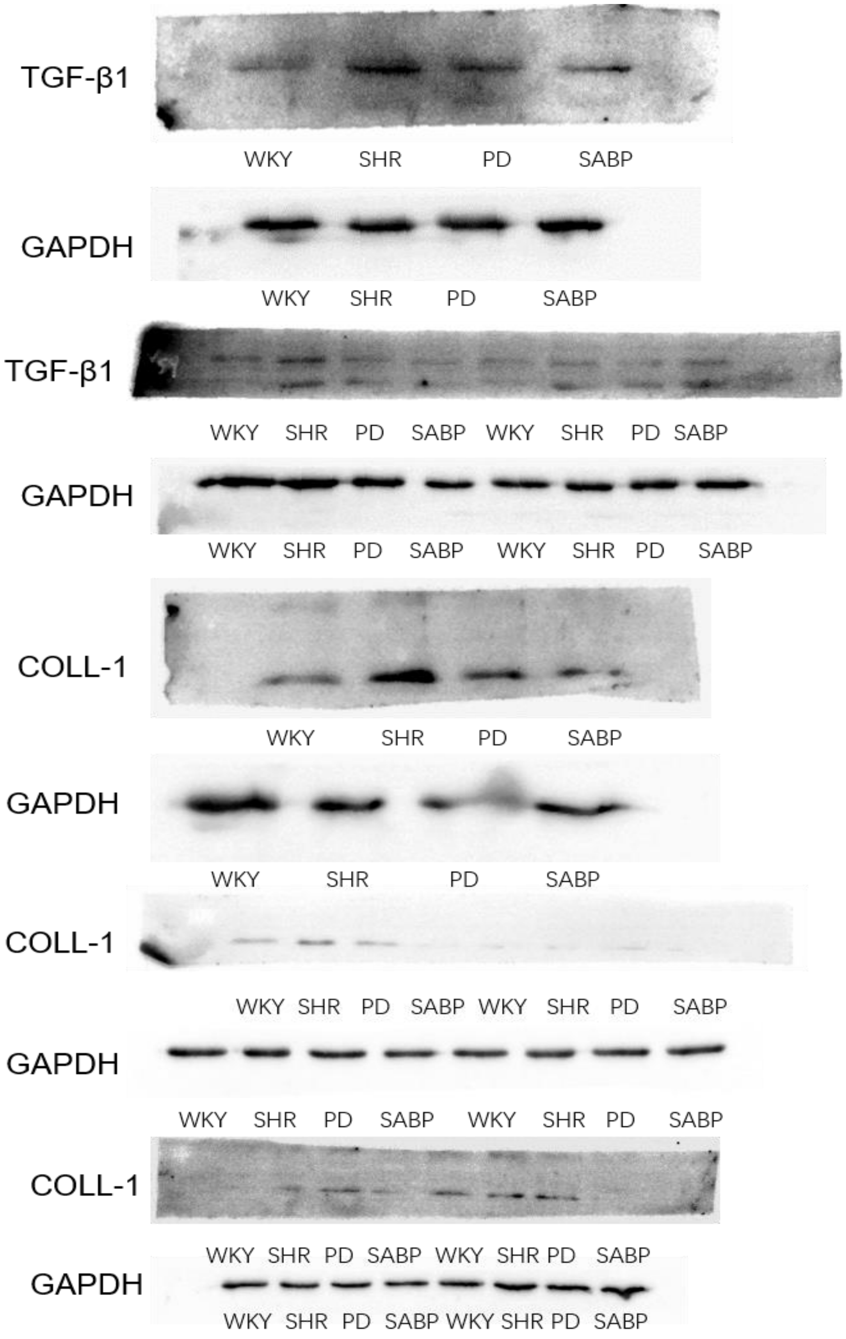

The above figures are the original western blot of vascular fibrosis detection indicators in the thoracic aorta of SHR in **Figure 6E** of the manuscript, including TGF-β and COLL-1. NAPDH is an internal reference control.

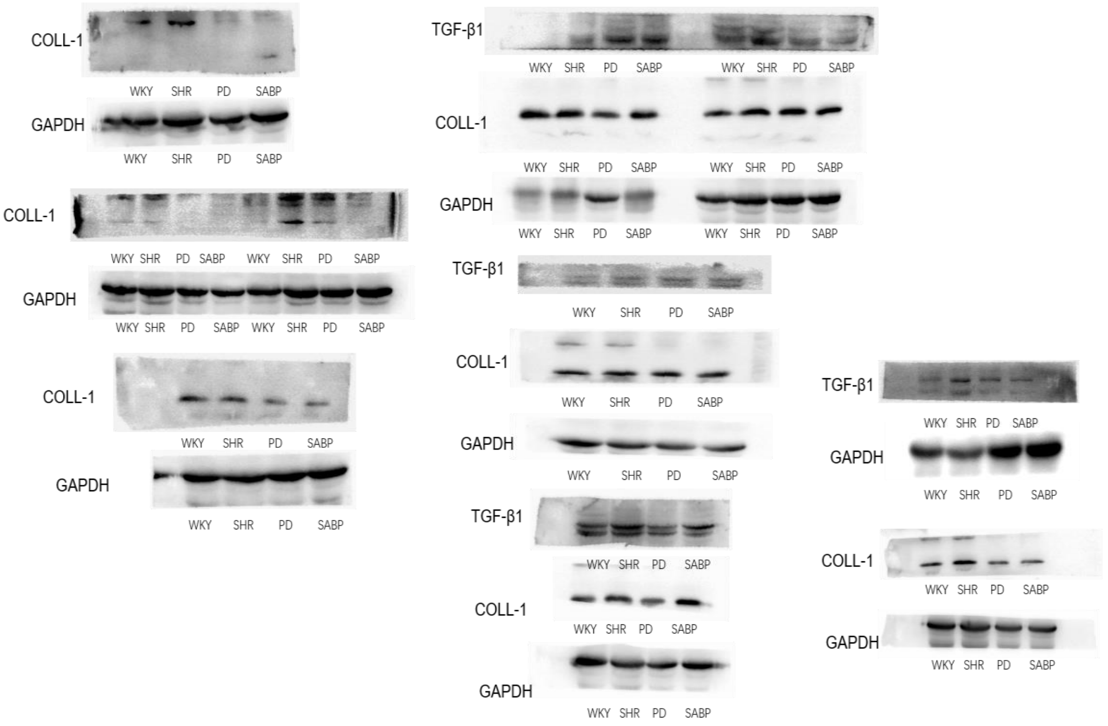

The above figures are the original western blot of the renal fibrosis detection indicators of SHR in **Figure 6G** of the manuscript, including TGF-β and COLL-1. NAPDH is an internal reference control.

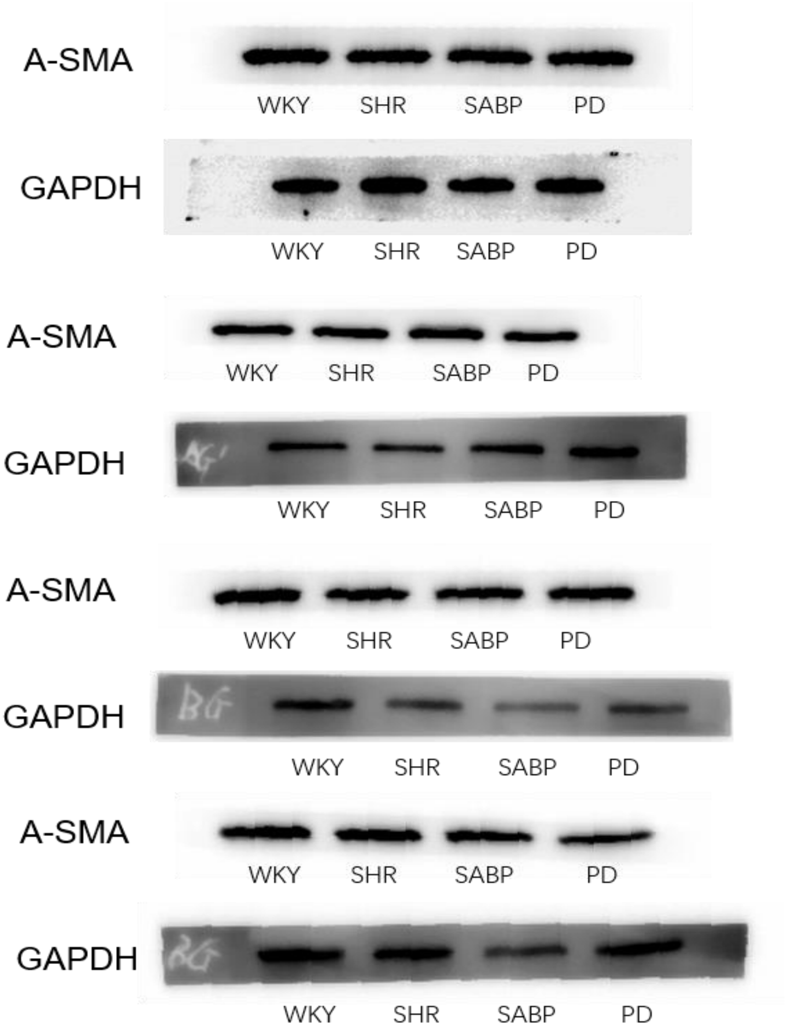

The above figures are the original western blot of transdifferentiation detection indicator (α - SMA) of thoracic aorta adventitia fibroblast of SHR in **Figure 7F** of the manuscript, with NAPDH as the internal reference control.

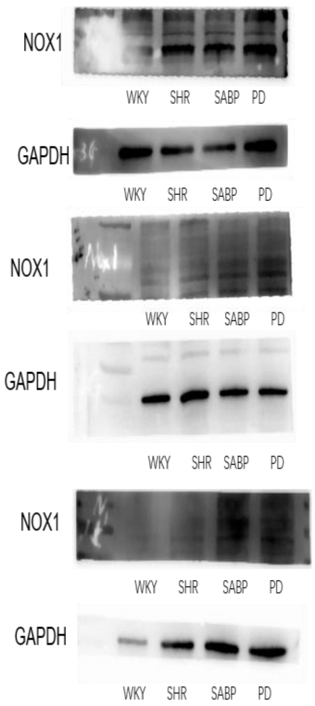

The above figures are the original western blot of oxidative stress detection index (NOX1) of thoracic aorta adventitia fibroblasts of SHR in **Figure 8A** in the manuscript, and GAPDH is used as the

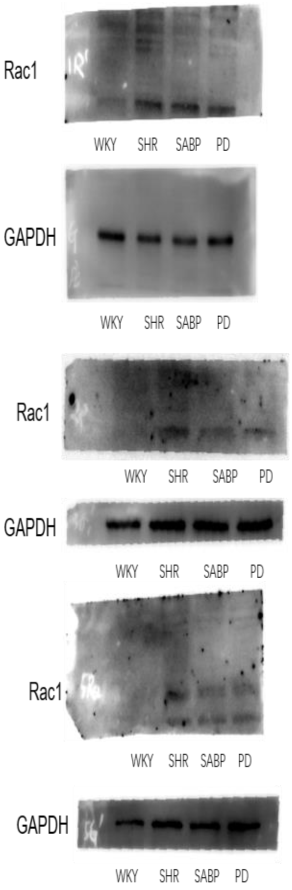

The above figures are the original western blot of mitochondrial damage marker protein Rac1 of thoracic aorta adventitia fibroblasts of SHR in **Figure 8C** in the manuscript, and GAPDH is used as the internal reference

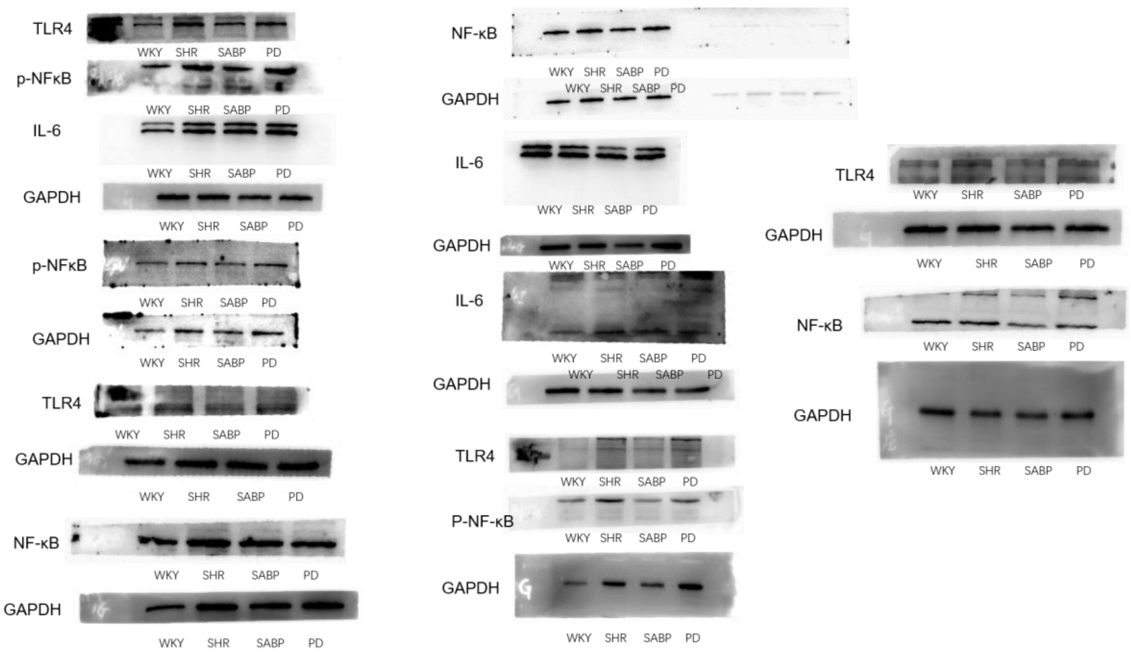

The above figures are the original western blot of inflammatory detection indicators of thoracic aorta adventitia fibroblasts of SHR in **Figure 8E** in the manuscript, including TLR4, NF- κ B, pNF- κ B and IL-6. GAPDH is used as the internal reference

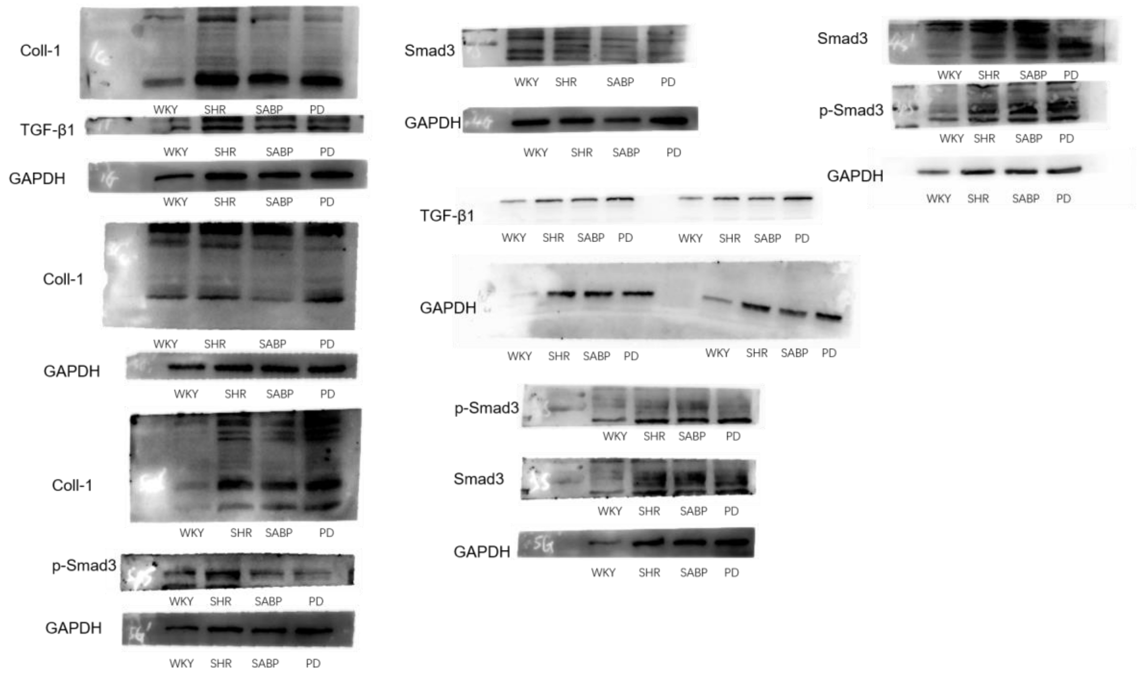

The above figures are the original western blot of fibrosis-associated protein detection of thoracic aorta adventitia fibroblasts of SHR in **Figure 8G** in the manuscript, including TGF-β , Smad3, pSmad3 and COL-1. GAPDH is used as the internal reference
